# Supplementary material for: Developmental loss of NMDA receptors results in supernumerary forebrain neurons through delayed maturation of transit-amplifying neuroblasts
Source: Sci Rep. 2024 Feb 9;14:3395. doi: 10.1038/s41598-024-53910-7 (PMC10858180; doi:10.1038/s41598-024-53910-7)
Supplement: Supplementary file 1 — Supplementary Figures. [file 41598_2024_53910_MOESM1_ESM.pdf]

Title: **Developmental loss of NMDA receptors results in supernumerary forebrain neurons through delayed maturation of transit-amplifying neuroblasts.**

Authors: Amalia J. Napoli[1], Stephanie Laderwager[1,2], Josiah D. Zoodsma[1], Bismi Biju[1], Olgerta Mucollari[1], Sarah K. Schubel[1], Christieann Aprea[1], Aaliya Sayed[1], Kiele Morgan[1], Annelysia Napoli[1], Stephanie Flanagan[1], Lonnie P. Wollmuth§[1,3,4], and Howard I. Sirotkin§[1]

Author Affiliations: [1]Dept. of Neurobiology & Behavior; Stony Brook University, 11794-5230
[2]Graduate Program in Neuroscience; Stony Brook University, 11794-5230
[3]Dept. of Biochemistry & Cell Biology; Stony Brook University, 11794-5215
[4]Center for Nervous System Disorders; Stony Brook University, 11794-5230
§ Co-senior authors

Address for correspondence: Dr. Howard I. Sirotkin
Depts. of Neurobiology & Behavior
Stony Brook University
Stony Brook, New York 11794-5230
Tel: (631) 632-4818
Fax: (631) 632-6661
E-mail: howard.sirotkin@stonybrook.edu

ORCID Numbers: Amalia J. Napoli 0000-0002-2603-8563
Stephanie Laderwager 0009-0002-6525-5613
Josiah Zoodsma 0000-0002-3014-860X
Olgerta Mucollari 0000-0003-3698-6409
Lonnie P. Wollmuth 0000-0002-8179-1259
Howard I. Sirotkin 0000-0002-1428-867X

Acknowledgements:

Research reported in this publication was supported by the National Institute of General Medical Sciences of the National Institutes of Health under Award Number K12GM102778 to A.J.N, the National Science Foundation Graduate Research Fellowship under grant Number 1839287 to A.J.N, a Stony Brook University Presidential Dissertation Completion Award to A.J.N., a Simons Summer Fellowship to K.M., Stony Brook University URECA summer fellowships to B.B and O.M., NIH grants to L.P.W (R01SNS088479), H.I.S and L.P.W (5R03HD101767-02). We thank Dr. James Napoli for his assistance with R applications. Special thanks to Wendy Akmentin for technical expertise and assistance, Dr. Alice Powers for critical comments on the manuscript, Dr. Bernadette Holdener and Dr. Mary Kritzer for technical advice, Dr. Shaoyu Ge for sharing reagents, and the many undergraduate students who assisted with fish care. The content of this publication is solely the responsibility of the authors and does not necessarily represent the official views of the National Institutes of Health, National Science Foundation, or Stony Brook University.

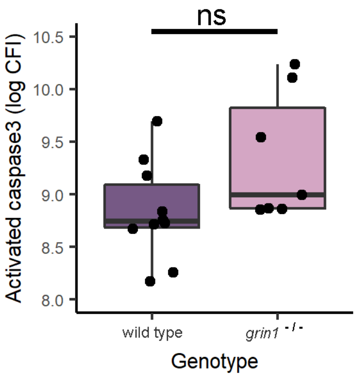

Supplementary Figure 1. Supernumerary neurons in the forebrain of *grin1*$^{-/-}$ fish do not result from a failure of programmed cell death.

CFI results for assay of activated caspase-3 in 3 dpf fish showing no statistical difference between the level of programmed cell death in wild-type and *grin1*$^{-/-}$ fish. Wild type n = 10, *grin1*$^{-/-}$ n = 7.

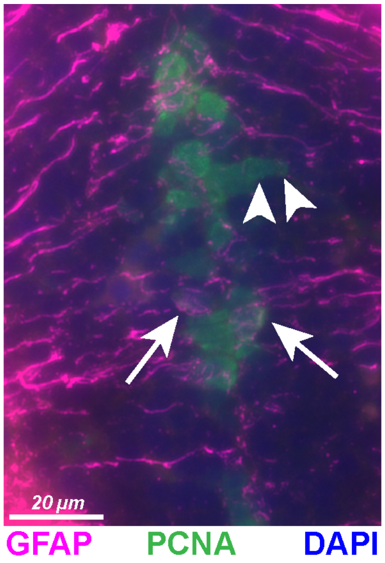

Supplementary Figure 2. Identification of radial glia and transit amplifying cells with GFAP and PCNA Expression

Wild-type 3 dpf fish expressing GFAP and PCNA with DAPI counterstain. Example $GFAP^{+}$ and $PCNA^{+}$ cells at PVZ designated as RGCs (arrows). Example $GFAP^{-}$ and $PCNA^{+}$ abventricular cells designated as tACs (arrowheads).
